# The National Insurance Scheme

**Published:** 1911-05-13

**Authors:** 


### SPECIAL INSTITUTIONAL ARTICLE.

### THE NATIONAL INSURANCE SCHEME.

The Chancellor of the Exchequer's scheme of national insurance, as expounded in his speech in the House of Commons, appears a colossal thing. The sickness scheme is twofold. There is the compulsory class of insurance and the voluntary class; the former is applicable only to employees, and then only if their wages are below £160 a year, while the latter applies to employees whose salaries exceed that sum and to persons not working for an employer. Since his speech Mr. Lloyd George has explained to Press representatives that there is no income limit for the voluntary contributor, and has expressed the opinion that many people "will think it worth while to join for the sake of the Government 2d. a week and the advantages of a well-organised and well-controlled scheme of State assistance in time of illness." This is tantamount to a proposal for the nationalisation of the medical service except in the case of the upper, upper middle, and wealthy classes. In examining the scheme it is therefore of supreme importance to keep in view the vast possibilities of that part of it which in effect provides that almost anybody, no matter what his income or his station in life may be, by paying 7d. per week may secure medical service during sickness. It is this part of the scheme which promises the most far-reaching effects. As to the compulsory class of insurance, the Bill mainly proposes to extend, improve, reorganise, endow, and make compulsory a system which now exists as a voluntary system. There are already over six millions of cases in which provision is made, for the most part through Friendly Societies, against sickness, mostly through an arrangement for medical aid. When insurance is made compulsory this number will, it is estimated, be more than doubled.

### The Government Proposals.

Briefly, the proposals may be outlined as follows: In the compulsory class, which includes employees earning less than £160 a year, the male worker is to pay 4d. a week out of his wages to the insurance fund, and the woman worker 3d. The employer in both cases is to pay 3d. a week and the State 2d. In the case of men working at low wages the rate of payment is to be less and the benefit less. In the voluntary class the person insured pays sevenpence instead of fourpence. In addition to free medical relief and free drugs, provision is made for sick allowances of 10s. a week for three months, and then 5s. a week up to the end of six months in the case of men, and 7s. 6d. and 5s. in the case of women; there will be a permanent disablement allowance of 5s. a week. Young people under sixteen will have no sick pay, but will get medical attendance; but persons over sixty-five will not be admitted to insurance. Consumption sanatoria are to be built throughout the country, and for this purpose it is proposed to set aside £1,500,000 as a capital sum for aiding local schemes; in order to provide maintenance it is proposed to take a contribution of 1s. a year per member for the whole of those who are insured compulsorily or voluntarily, to which the State will add 4d., so that there will be 1s. 4d. per member for the purpose of raising a fund for the maintenance of these institutions. Further, there is to be a maternity grant of 30s. to mothers, whether married or unmarried, but it is not clear that this 30s. is going to the practitioners and the nurse. Mr. Lloyd George confessed that he had experienced some difficulty in fixing the standard of medical fees. He admitted it was not right "that we should do our charity at the expense of a hard-worked profession," and he agreed that "the doctors have got a case for increased payment." The sum he suggested in his speech, however, 4s. per head per annum, falls far short of the standard at which practitioners have been aiming, and is considerably less than is already paid by some societies; but he has since stated that he hoped to fix a higher rate. To relieve the practitioner of the cost of drugs and the work of dispensing, which it is proposed to do, cannot be regarded as sufficient compensation for the smallness of the fee suggested. So far there is no reference to provision for hospital attendance, but in due course the position of these institutions will, no doubt, be explained; for who is to perform operations, and where are they to be done?

### Administration.

The bulk of the work of administration will fall upon the "approved" Friendly Societies, but there is to be a Central Insurance Board, upon which medical men will be represented. By "approved" Friendly Society is meant one which has a membership of at least 10,000; it must be precluded by its constitution from distributing any of its funds otherwise than by way of benefits among its members, and it must not be a dividing society. All the men in a county who have not joined a society, however, are to be collected together in a body called Post Office contributors, and this part of the scheme is to be administered differently—namely, by county health committees, whose members are to be chosen in three batches, a third by the county councils, a third by the approved societies in the districts, and a third by the Post Office insurance, and the State will also have a fourth of the whole body to represent its interests. The county health committees are also to administer the whole of the sanatorium funds. Briefly, these are the main proposals, but the scheme is so comprehensive that even some of the subsidiary proposals are of an extremely important character, and demand equally careful consideration. The scheme embodies provisions not only for the relief of sickness but for the prevention of sickness; for it is proposed that whenever there is excessive sickness a county health committee shall have power to go to the Local Government Board and apply for an inquiry into its cause. Wherever the Commissioners of the Board find that it is due to neglect by the authority to discharge functions imposed by an Act of Parliament for the housing of the people, or for improved sanitation, it is provided that they shall have power of imposing the consequent excess in expenditure, not on the society, but upon the local authorities which are at fault. The sums paid by all classes of contributors in the first year will, it is estimated, amount to nearly £20,000,000, of which the employers will contribute nearly £9,000,000 and the employees £11,000,000. It will be observed that the difficulty which was at first thought to stand in the way of such a scheme as that here outlined—namely, the opposition of the friendly societies as representing powerful vested interests—has been overcome by utilising the friendly societies as part of the very scheme which it had been feared would crush them. As to the medical profession, it is not without interest to quote from a book which was recently reviewed in The Hospital; in "The Dawn of the Health Age" Dr. Benjamin Moore, in outlining a scheme of national medical service, says: "To turn the whole of the present medical men practising in the country immediately into salaried medical officers would prove impossible. There exists no basis, to begin with, upon which the salaries of the doctors could be calculated in a trustworthy way, and such a basis must first be obtained. For the first period of running of the scheme, then, it would have to be thrown open to the whole of the medical profession, and payment would have to be made upon the basis of some scheme such as one or other of several plans, differing in detail, which have been discussed by the British Medical Association and other bodies." The position of medical practitioners under the scheme now under consideration will become clearer after a careful examination of the text of the Bill which has only just been circulated. The fact that provision is made not only for medical attendance on an insured person but also on persons dependent upon his labour will have to be taken into account when the question of medical fees comes up for consideration. The scheme is the beginning of something which promises, in course of time, to have an enormously important influence on the practice of medicine in this country.

